# Key Role of Water-Insoluble Allergens of Pollen Cytoplasmic Granules in Biased Allergic Response in a Rat Model

**DOI:** 10.1097/WOX.0b013e318205ab44

**Published:** 2011-01-15

**Authors:** Oussama R Abou Chakra, Jean-Pierre Sutra, Pascal Poncet, Ghislaine Lacroix, Hélène Sénéchal

**Affiliations:** 1 ESPCI, ParisTech, LECA, Paris, France; 2 Institut Pasteur, Infection and Epidemiology, Paris, France; 3 INERIS, DRC, Verneuil-en-Halatte, France; 4 INSERM, CSS 5, Paris, France

**Keywords:** allergenicity, grass pollen, IgE, pollen cytoplasmic granules, water-insoluble allergens, water-soluble allergens

## Abstract

**Background:**

Grass pollen grain, an important aeroallergen, can disperse in the environment pollen cytoplasmic granules (PCGs) able to release water-soluble allergens when they are washed out by rainfall. The allergenicity of these washed PCGs is, however, preserved.

**Objective:**

The purpose of the study was to assess the allergenic potential of washed and unwashed PCGs, from *Phleum pratense *pollen grains, in the Brown Norway rat, and to study the IgE reactivity of sera of sensitized rats to water-soluble and water-insoluble extracts of PCGs and pollen grains.

**Methods:**

Rats were sensitized and challenged intratracheally with washed or unwashed PCGs or pollen grains. Using water-soluble and -insoluble extracts of pollen grains and/or PCGs, IgE ELISA and immunoblotting were performed with rat sera. Proliferation of bronchial lymph node cells was monitored by [^3^H]-thymidine incorporation in a lymph node assay. Alveolar cells, proteins, and T_H_1 and T_H_2 cytokines were quantified in bronchoalveolar lavage fluid.

**Results:**

Rats sensitized with unwashed PCGs showed a predominant humoral response with high serum IgE and reactivity to water-soluble and -insoluble proteins together with low lymph node cell proliferation. Conversely, in rats sensitized to washed PCGs, cellular responses were higher with significant increases in eosinophils, lymphocytes, and T_H_2 cytokines observed in bronchoalveolar lavage fluid.

**Conclusion:**

Allergic and inflammatory responses were induced by both grass pollen grains and their isolated washed and unwashed PCGs. However, on the basis of humoral and cellular responses, differential patterns were observed. Water-insoluble allergens seem to play a role in the centrally mediated inflammatory response, whereas water-soluble allergens may be involved in the peripheral humoral response.

## 

Grass pollen is quantitatively one of the most important aeroallergen vectors worldwide. It is a major cause of allergic reactions including conjunctivitis, rhinitis, and other upper and lower respiratory tracts problems occurring during the flowering season of different grasses. During the last 40 years, the frequency of symptoms of allergic diseases has increased dramatically, especially in children and people living in urban areas. Several factors have contributed to this increase, among which airborne pollutants--from gaseous and particulate emissions--are growing in importance [1].

Airborne pollen grains can release hundreds of small particles called pollen cytoplasmic granules (PCGs) [2,3]. These may be present in atmospheric samples taken during the pollen season, and some studies show a 50-fold increase in their atmospheric concentration on days after rainfall [3,4]. In the same way, airborne pollutants may modify pollen grains structurally, thereby increasing the release of PCGs in the atmosphere [5].

Because PCGs are small (< 3 *μ*m in diameter) and occur at high atmospheric concentrations, in particular, on days after rainfall, several research groups have studied their allergenic potential. PCGs elicit IgE-mediated responses in asthmatic patients and induce bronchial constriction in patients with rainfall-associated asthma [6]. In vivo studies show that PCGs, and pollen grains, induce humoral and cellular responses in animal models of allergy [7-10]. Furthermore, in vitro, PCGs increase inflammatory responses in bronchial epithelial human cells and rat macrophages [8,11]. All these studies were performed with washed PCGs to preserve only PCG allergens. However, both washed and unwashed PCGs can be present in natural atmospheric conditions, depending on rainfall.

In molecular allergy studies, several water-soluble pollen allergens have been found in PCGs by immunogold labeling and Western blot studies, such as Lol p 1b and Lol p 5 for rye grass, Phl p 4, Phl p 5, Phl p 6, and Phl p 13 for Timothy grass [12-16]. In urban environments, water-soluble allergens may be extracted by rain from either pollen grains or PCGs, leaving in the particles mostly water-insoluble proteins, the allergenicity of which has been little studied, if at all. However, some water-insoluble proteins have been reported to be major allergens, in plant parts other than pollen grains, such as gliadin in wheat[17] and Hev b 1 and Hev b 3 from natural rubber [18].

The purpose of our work was to provide data on the allergenicity of both washed and unwashed PCGs from *Phleum pratense *pollen grains in an experimental animal model. These experiments were performed in the Brown Norway rat, a good model of allergy[19-22] that we have used for several years, [7,9,10] by intratracheal instillation, a simple and rapid technique allowing the introduction of a controlled dose of the allergenic product.

Humoral and cellular allergic responses were studied. Serum IgE specificities to water-soluble and water-insoluble pollen grains and/or PCGs were evaluated by enzyme-linked immunosorbent assay (ELISA) and 1D immunoblotting. Isoelectric focusing (IEF) was used to analyze separated native proteins from grass pollen, leading to well-known electrophoretic patterns [23]. Cellular responses were assessed by lymph node assay, by counts of eosinophils and lymphocytes in bronchoalveolar lavage fluid (BALF), and by quantitation of T_H_2 cytokine in BALF. Depending on the sensitizing agent studied, differential response patterns were observed.

## Materials and methods

### Animals

Male Brown Norway (BN) rats were obtained from Charles River Laboratories (France). All rats were 6 to 8 weeks old when starting the experiments. Animals were housed in the INERIS animal care facility and had access to conventional laboratory feed and water. Rats were handled in accordance with French and European State Council guidelines for the care and use and ethical treatment of laboratory animals (Decree 2001-486187 and directive 86/609/EEC, respectively). The experiments were approved by the INERIS Institutional Animal Care and Use Committee. The rats were randomly divided into 4 groups of 6 rats each.

### Pollen and PCGs

Pollen grains from Timothy grass (*Phleum pratense*) were collected at Alk Abello (Varennes-en-Argonne, France) and immediately sent to our laboratory after the harvest. This pollen was not treated postharvest and was kept in optimal conditions (4°C).

Pollen grains (15-60 *μ*m, average diameter = 31 *μ*m) suspended in saline solution were used to immunize the rats.

The water-soluble pollen extract (ws-Pol) was prepared according to Mahler et al [24] for IgE ELISA or to Rogerieux et al [25] for IEF. The water-insoluble pollen extract (wi-Pol) was prepared according to Godfrin et al [26].

PCGs (0.6-5 *μ*m, average diameter = 1.1 *μ*m) were isolated from *Phleum pratense *pollen by osmotic shock in pure water [27]. The size and number of PCGs were determined with a particle counter (Z3 Multisizer Coulter Counter, Beck-man Coulter). Unwashed PCGs were obtained after filtration, using Ultrafree-CL Centrifugal Filter Unit (Millipore), and centrifugation of the initial material.

Washed PCGs were obtained by filtration and centrifugation and were then washed two times in distilled water. The water-soluble protein concentrations in the suspensions of washed and unwashed PCGs were about 4 and 80 *μ*g/mL, respectively. Washed and unwashed PCGs were resuspended in saline solution for immunization of the rats.

Endotoxins/lipopolysaccharides (LPS) were detected in pollen and unwashed PCGs using the *Limulus amebocytes *lysate assay (E-Toxate Kit, Sigma). LPS were not detected in washed PCGs.

The water-soluble PCG extract (ws-PCGs) corresponds to the supernatant of the second wash of PCGs. Before IEF, this extract was concentrated tenfold.

The water-insoluble PCG extract (wi-PCGs) was obtained after 6 extensive washes of PCGs. The pellet was resuspended in a TUC mixture: 2 M thiourea, 7 M urea, 5% (wt/vol) 3-[(3-cholamidopropyl) dimethylammonio]-1-propanesulfonate (CHAPS). PCG extracts (ws-PCGs and wi-PCGs) were both used for IEF.

### Sensitization, Challenge, and Autopsy of the Rats

Rats were immunized following the protocol already described [7]. Briefly, rats were anesthetized, and with use of a cannula, a suspension of allergens was intratracheally instilled on day 0. This was repeated on day 21 for the challenge. The 3 allergens used were pollen grains (0.5 mg per rat), washed PCGs (4.5 × 10^7 ^per rat), and unwashed PCGs (4.5 × 10^7 ^per rat). Saline solution was instilled in negative control (NC) rats.

Rats were killed 4 days after challenge. Blood samples, BALF, and bronchial lymph nodes were collected.

### Sera

#### Rat serum

After collection, rat blood samples were kept at 4°C to allow clotting (2-4 hours) and then centrifuged for 10 minutes at 2000 *g *and 4°C. Serum was collected and stored in 500-*μ*L aliquots at -80°C until use.

#### Human serum

From our bank of patient sera, one serum (no. 15) was selected from a group of 26 donors allergic to grass pollen for its ability to recognize a great number of grass pollen allergens after ELISA and blotting [23]. This serum was used for immunoblotting after IEF.

### Enzyme-Linked Immunosorbent Assay for Specific Anti-Timothy Pollen IgE

The ELISA was performed in serum, using ws-Pol and wi-Pol, according to the pollen-dioxygenin protocol previously described, [10] with slight modifications. Briefly, 1:500 diluted mouse anti-rat IgE antibodies (Zymed Laboratories) were used to coat 96-well microtitre plates. After washing and saturation of the wells with 1% (wt/vol) skimmed milk powder solution, rat sera were added to the wells (in duplicate) at the proper dilution (1:2) and incubated for 1 hour at 37°C. After washing, wells were incubated with the pollen extracts (ws-Pol and wi-Pol) (dilution 1:800), and after a washing step, a horseradish peroxidase-conjugated antidioxygenin antibody was added (dilution 1:625, Roche Diagnostics). The amount of IgE was measured by adding peroxidase substrate and reading the absorbance values at 450 nm with a multichannel photometer (Tecan Group Ltd.).

### Isoelectric Focusing and Immunoblot

IEF separation of pollen and PCG extracts was performed in a polyacrylamide gel (CleanGel IEF, GE Health-care, Bio-Sciences AB) containing 5% vol/vol Servalyt pH 2-11 (Serva Electrophoresis GmbH), in water or TU mixture (2 M thiourea, 7 M urea) for water-soluble and water-insoluble extracts, respectively. According to the manufacturer's instructions, the flat bed electrophoretic chamber (Multiphor II, GE Healthcare, Bio-Sciences AB) was cooled at 15°C for water-soluble extracts (ws-Pol and ws-PCGs) and 18°C for water-insoluble extracts (wi-Pol and wi-PCGs). After the protein separation, a part of the gel was stained with Coomassie Blue. Isoelectric point (pI) standards from 4.45 to 9.6 (Bio-Rad Laboratories) were used as references.

After IEF separation, proteins of each extract were blotted by pressure (for 1 hour at 22°C) onto a polyvinylidene fluoride sheet (pore size, 0.2 *μ*m; Immobilon, Millipore). Polyvinylidene fluoride sheets were then cut into strips (2.5 mm wide), saturated in skimmed milk powder solution (5% wt/vol in phosphate-buffered saline [PBS]-0.1% Tween) for 1 hour at room temperature and then individually incubated for 1 hour with rat sera or human sera. In rats, 3 serum samples were used in each experimental rat group (dilution 1:5 and an additional dilution 1:100 for wi-Pol). Strips were then incubated with a mouse anti-rat IgE antibody (dilution 1:500; Zymed Laboratories) followed by an alkaline phosphatase-conjugated goat anti-mouse IgG antibody (Sigma Chemical). For the human serum (dilution 1:10), strips were incubated with an alkaline phosphatase-conjugated goat anti-human antibody (dilution 1:700; Sigma Chemical). Finally, all strips were revealed with alkaline phosphatase substrates, 5-bromo-4-chloro-3-indolyl phosphate and nitroblue tetrazolium.

### Bronchoalveolar Lavage Fluid

Lungs were flushed in situ 3 times with 10 mL of a sterile PBS solution (pH 7.2). Fluid collected from the bronchoalveolar lavage of each rat was centrifuged at 150 *g *for 10 minutes. Pellets were used to count alveolar cells and supernatants were used for quantitation of proteins and cytokines.

### BALF Alveolar Cells

The collected alveolar cells (9-20 *μ*m) were counted with a Z2 Coulter Counter (Beckman Coulter) and applied to a slide by centrifugation using a Shandon Cytospin 2 at 150 *g *for 5 minutes. Cell differential counts were performed after May-Grünwald-Giemsa staining and a minimum of 400 cells were counted per slide. Macrophages, eosinophils, lymphocytes, and neutrophils were identified in BALF.

### Proteins and Cytokines in BALF

Cell-free BALF was concentrated using Amicon Ultra-15 Centrifugal Filter Units (Millipore) until the volume was equal to 1 mL. The protein concentrations were determined by the Lowry method [28].

Cytokines were quantified in concentrated BALF using, first, the Bio-Plex kit for IL-1*α *(interleukin-1*α*), IL-1*β*, IL-2, IL-4, IL-6, IL-10, Granulocyte Macrophage - Colony Stimulating Factor (GM-CSF), Interferon-*γ *(IFN*γ*), and Tumor Necrosis Factor-*α *(TNF*α*) (catalog no. 171K11070, Bio-Rad Laboratories) and, second, the rat cytokine multiplex immunoassay kit for IL-5, IL-13, Eotaxin, and Regulated upon Activation, Normal T-cell Expressed, and Secreted (Rantes) (or Chemokine (C-C motif) Ligand 5 [CCL5]) (catalog no. RCYTO-80k-04, Linco, Millipore), according to the manufacturer's instructions.

### Bronchial Lymph Node Cell Assay

The bronchial lymph node assay was previously described [7]. Different allergens (pollen, 10 *μ*g/mL; washed PCGs, 9 × 10^5 ^PCGs per mL; unwashed PCGs, 9 × 10^5 ^PCGs per mL) were then added in vitro to bronchial lymph node cells.

### Statistical Analysis

The results of all studied parameters are expressed as means ± SEM. All statistical analyses were performed with SPSS (version 11.5, Lead Technologies Inc.) using nonparametric tests (Kruskall-Wallis and Mann-Whitney). Statistical significance was defined with *P <*0.05 (two-tailed).

## Results

### Pollen-Specific IgE Levels in Rat Sera (Table 1)

Levels of IgE antibody directed against water-soluble (ws) and water-insoluble (wi) pollen extract were measured by ELISA in sera of rats immunized with washed PCGs or unwashed PCGs or pollen grains (Table 1). As compared with the NC group, IgE antibodies were found, except to wi-pollen extract, in the sera of rats sensitized with washed PCGs. The order of IgE Ab response was the same for ws-pollen and wi-pollen extracts: unwashed PCGs > pollen grains > washed PCGs. Interestingly, 5 to 6 times more IgE antibodies were revealed with ws-pollen than with wi-pollen extract.

**Table 1:** Rat Serum Levels of IgE Specific to Timothy Grass (Mean ± SEM)

Treatment Group	NC	Washed PCGs	Unwashed PCGs	Pollen
ws-Pol	0.08 ± 0.002	0.38 ± 0.12*†	2.73 ± 0.23*†	0.93 ± 0.11*
wi-Pol	0.05 ± 0.01	0.08 ± 0.02†	0.44 ± 0.06*	0.15 ± 0.04*†

IgE absorbance (at 450 nm) was measured by ELISA, using water-soluble (ws) and water-insoluble (wi) pollen extracts. NC, negative control group; pollen, rats sensitized and challenged with 0.5 mg of pollen; unwashed PCGs, rats sensitized and challenged with 4.5 × 10^7 ^unwashed PCGs; washed PCGs, rats sensitized and challenged with 4.5 × 10^7 ^washed PCGs.

*Significantly different from NC (*P <*0.05).

†Significantly different from pollen group (*P <*0.05).

### IgE Reactivity To Proteins From Different Extracts

To study the diversity of the repertoire of recognized allergens, IgE Ab binding was assessed by Western blotting with water-soluble and -insoluble proteins from pollen and PCGs separated by IEF. For all extracts, serum samples from rats sensitized to unwashed PCGs presented higher reactivity than all other rat sera groups.

Although each rat expressed a specific quantitative and qualitative IgE response, a similar overall pattern of recognition may be distinguished between rats sensitized with the same allergenic source.

### Water-soluble pollen extract (Figure 1A)

Several IgE-binding proteins (approximately 20 bands covering the whole pI range) were observed with sera (diluted 1:5) from rats sensitized to washed PCGs (strips 4-6), unwashed PCGs (strips 7-9), and pollen (strips 10-12). Patterns of recognition were very similar, although not identical, between the different types of sensitization. However, the intensity of IgE reactivity was highest in rats sensitized to unwashed PCGs (strips 7-9) and lowest in rats sensitized to washed PCGs (strips 4-6). For the NC group (strips 1-3), a very faint signal was observed.

**Figure 1:**
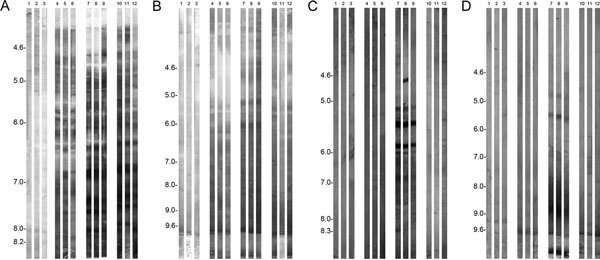
**The IgE binding protein of pollen or PCG extracts separated by IEF followed by immunoblotting with rat sera**. A, Water-soluble pollen extract; B, water-insoluble pollen extract; C, water-soluble PCG extract; D, water-insoluble PCG extract. Each strip was incubated with rat serum: strips 1-3, negative control group; strips 4-6, rats sensitized and challenged with 4.5 × 10^7 ^washed PCGs; strips 7-9, rats sensitized and challenged with 4.5 × 10^7 ^unwashed PCGs; strips 10-12, rats sensitized and challenged with 0.5 mg of pollen (pollen).

### Water-insoluble pollen extract (Figure 1B)

Several IgE reactive proteins were revealed, covering the whole pI range with sera (1:100) from the 3 groups of sensitized rats. Again, the intensity of IgE reactivity was the highest in rats sensitized to unwashed PCGs. Diffuse IgE reactivity was observed, in the basic area (pI ~ 7.5-9.6), for all groups of sensitized rats. For the NC group, no IgE binding reactivity was exhibited (strips 1-3).

### Water-soluble PCG extract (Figure 1C)

Proteins of this extract were not bound by IgE of sera from rats sensitized to washed PCGs (strips 4-6). About 12 protein bands (pI 4.5-8.3) were revealed by sera from rats sensitized to unwashed PCGs (strips 7-9). Interestingly, strong reactivity to an acidic protein (pI ~ 4.5) was noted in strip no. 8. For the pollen-sensitized rats (strips 10-12), only one serum (strip no. 12) showed a low IgE reactivity (protein bands with pI ranging between 5.6 and 7.0). No IgE binding reactivity was observed for the NC group (strips 1-3).

### Water-insoluble PCG extract (Figure 1D)

For sera from rats sensitized to washed PCGs (strips 4-6), only one strip (no. 6) showed low IgE binding reactivity to a protein with basic pI (~8.2). Using sera from rats sensitized to unwashed PCGs (strips 7-9), 2 IgE binding proteins (pI ~ 4.9 and 5.2) and a diffuse IgE reactivity, in the basic area (pI ~ 7.5-9.6), were observed. For the pollen-sensitized rats (strips 10-12), low reactivity was noted. No IgE binding reactivity was exhibited for the NC group (strips 1-3).

Taken together, Western blot reactivities were stronger with sera from rats sensitized to unwashed PCGs, thus confirming the IgE quantitation obtained by ELISA.

### Comparison between human and rat IgE reactivities (Figure 2)

Human IgE patterns were compared with those obtained with sera from rats sensitized to unwashed PCGs. For all extracts, a large number of proteins, with the same isoelectric points, were detected by rat IgE and with human IgE.

**Figure 2:**
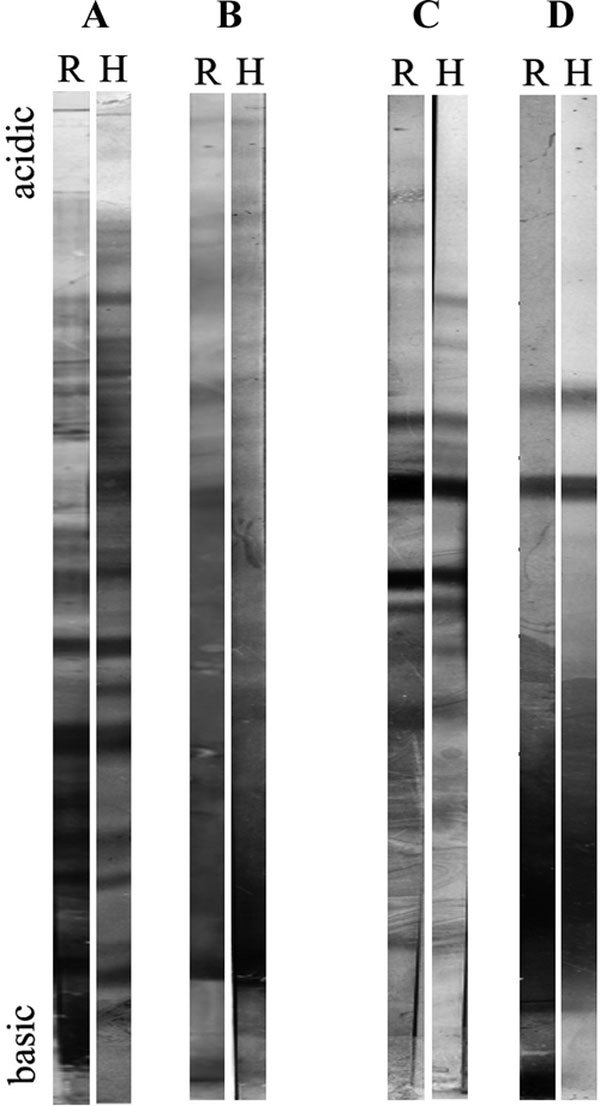
**The IgE binding proteins of pollen or PCG extracts separated by IEF followed by immunoblotting with rat (R) and human (H) sera**. A, Water-soluble pollen extract; B, water-insoluble pollen extract; C, water-soluble PCG extract; D, water-insoluble PCG extract.

### Protein and Cytokines in BALF

Local inflammation of the lung was explored by evaluating total protein and cytokine concentrations in BALF from sensitized and unsensitized rats.

### Protein concentrations

All sensitizations induced a threefold rise in protein concentration as compared with the NC group (1.85 versus 0.68 ± 0.09 mg/mL).

### Cytokine concentrations (Figure 3)

Washed PCGs--that is, granules containing mainly water-insoluble proteins--were the strongest inducer of proallergy cytokines in BALF: IL-5 (approximately ×17), IL-13 (approximately ×3), and Rantes (approximately ×3). Rantes was also increased threefold in BALF of rats sensitized with unwashed PCGs. Pollen had a moderate effect on proallergic cytokine production.

**Figure 3:**
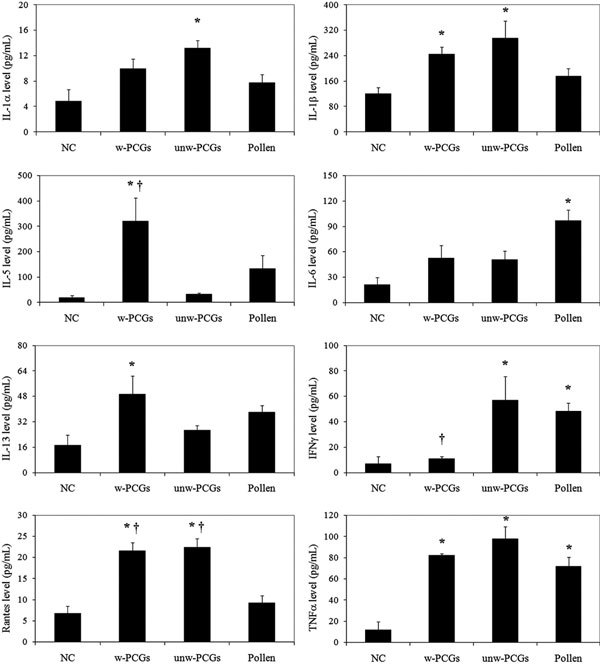
**BALF cytokine levels (mean ± SEM)**. Among proallergy cytokines, IL-5, IL-13, and Rantes were quantified. Among proinflammation cytokines, IL1*α*, IL-1*β*, IL-6, IFN*γ*, and TNF*α *were quantified. NC, negative control group; pollen, rats sensitized and challenged with 0.5 mg of pollen; unw-PCGs, rats sensitized and challenged with 4.5 × 10^7 ^unwashed PCGs; w-PCGs, rats sensitized and challenged with 4.5 × 10^7 ^washed PCGs; *, significantly different from NC (*P <*0.05); **†**, significantly different from the pollen group (*P <*0.05).

Except for IL-6, unwashed PCGs were the strongest inducers of proinflammatory cytokines: IL-1*α *(approximately ×3), IL-1*β *(approximately ×2.5), IFN*γ *(approximately ×8), and TNF*α *(approximately ×8). The highest increase in IL-6 was observed upon pollen sensitization. No IFN*γ *was found in BALF from rats sensitized to washed PCGs. Interestingly, the 3 immunizations induced high expression of TNF*α*, likely reflecting production of various cells.

The other cytokines, IL-2, IL-4, IL-10, eotaxin, and GM-CSF, were either undetectable or at very low concentrations in the BALF of all rat groups (data not shown).

### Alveolar Cells in BALF (Table 2)

The 3 immunizations induced eosinophil recruitment in BALF, whereas lymphocytes only increased upon PCG sensitization. With regard to proinflammatory cells, whatever the sensitization, macrophages were recruited at a similar level. Neutrophil numbers increased.

**Table 2:** Alveolar Cells (Mean ± SEM) in BALF

Treatment Group	Eosinophills	Lymphocytes	Macrophages	Neutrophills
NC	0.40 ± 0.09	0.21 ± 0.06	3.4 ± 0.4	0.03 ± 0.01
Washed PCGs	5.32 ± 1.33*	0.63 ± 0.21*†	7.0 ± 1.6*	0.29 ± 0.10*
Unwashed PCGs	5.05 ± 1.18*	0.35 ± 0.12*	7.5 ± 0.4*	0.55 ± 0.21*
Pollen	400 ± 0.83*	0.11 ± 0.09	7.9 ± 1.3*	0.25 ± 0.11*

Alveolar cells were counted after May-$\text{Gr}\ddot{\text{n}}\text{wald}$-Giemsa staining. NC, negative control group; pollen, rats sensitized and challenged with 0.5 mg of pollen; unwashed PCGs, rats sensitized and challenged with 4.5 × 10^7 ^unwashed PCGs; washed PCGs, rats sensitized and challenged with 4.5 × 10^7 ^washed PCGs.

*Significantly different from NC (*P *< 0.05).

†Significantly different from the pollen group (*P *< 0.05).

Rats sensitized with unwashed PCGs had higher neutrophil numbers than rats in the pollen group, but this difference was not statistically significant.

### Lymph Node Cell Proliferation Induced by Pollen or PCGs (Figure 4)

**Figure 4:**
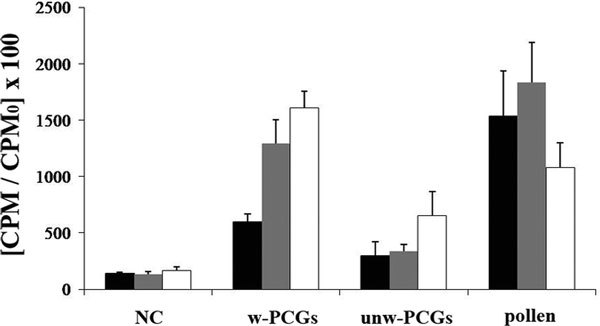
**Pollen-and PCG-induced proliferation of lymph node cells**. Cells from NC, pollen-sensitized, or PCG-sensitized rats were cultured with [^3^H]-thymidine in the presence of 10 *μ*g of pollen (black histograms), 9 × 10^5 ^unwashed PCGs (gray histograms), and 9 × 10^5 ^washed PCGs (white histograms). CPM, thymidine incorporation by antigen-stimulated cells; CPM0, thymidine incorporation in control cells; NC, negative control group; pollen, rats sensitized and challenged with 0.5 mg of pollen; unw-PCGs, rats sensitized and challenged with 4.5 × 10^7 ^unwashed PCGs; w-PCGs, rats sensitized and challenged with 4.5 × 10^7 ^washed PCGs. All proliferative responses were significantly different from their respective NC value (*P *< 0.05), except for rats sensitized to unwashed PCGs and challenged with pollen grains.

To further analyze the cellular response, T-cell precursor frequency was estimated by measuring the proliferative response of bronchial lymph node cells upon in vitro challenge with the different immunogens (Figure 4).

Pollen-sensitized rats were highly reactive to in vitro challenges with the 3 immunogens, suggesting a rather high frequency of T-cell precursors for all specificities, water-soluble and water-insoluble antigens. However, the highest proliferative response to washed PCGs, that is, water-insoluble antigens, was observed in rats sensitized to washed PCGs, whereas challenge with pollen, containing both water-soluble and -insoluble antigens, was a poor inducer of cellular proliferation. Rats sensitized to unwashed PCGs, although poorly reactive to the 3 in vitro challenges, showed a higher T-cell proliferative response to water-insoluble antigens (washed PCGs).

## Discussion

Pollen grains are complex biologic particles composed of membranes that protect the protoplasm, that is, cytoplasm, cytoplasmic granules (PCGs), and nucleus. Hydration of the pollen grains results in the release of water-soluble components and granules that are composed of mainly carbohydrates but also proteins. These PCGs play a key role in allergic sensitization because they (i) carry allergens, (ii) are small and can go deeper into the respiratory tract than pollen grains, and (iii) are continuously present in the atmosphere with sharp peaks after rainfall.

Most studied allergens are water-soluble proteins. However, pollen grains and their subfractions PCGs also include water-insoluble proteins, the proportion of which increases progressively with washing. In this study, allergenicity of washed and unwashed PCGs, from Timothy grass pollen, was assessed in Brown Norway rats and compared with the allergenicity of whole pollen grains. Humoral and cellular responses reported herein can be compared because the 3 groups of rats received, by intratracheal instillation, similar doses of immunogenic material. Therefore, differences in allergic and inflammatory responses observed should be attributed to qualitative differences in antigens and allergens. Washed PCGs contain mainly water-insoluble proteins and also a small amount of residual water-soluble proteins. Unwashed PCGs and pollen grains contain both water-soluble and -insoluble proteins differentially accessible to the immune system because of differences in assembly and exposure within the respective particles.

Quantitation of IgE levels by ELISA showed that water-soluble allergens are the main trigger of humoral responses, as previously suggested in sensitized rats [27]. Higher IgE levels were observed in rats sensitized by unwashed PCGs, as compared with pollen-sensitized rats, although water-soluble proteins should theoretically be at similar concentrations in both sensitizing allergens. This may be attributed to a higher accessibility of antigens in unwashed PCGs. In fact, we can calculate that 4.5 × 10^7 ^PCGs had a contact area with alveolar cells at least twice that corresponding to 0.5 mg of pollen grains. The correlation also applied to rats sensitized to washed PCGs, which had a low level of serum IgE because there are only trace amounts of water-soluble allergens in washed PCGs.

By using immunoblotting, we have shown that the IgE elicited by immunization with washed PCGs were highly reactive to allergens from pollen and barely reactive to proteins from PCGs. To obtain IgE antibodies to PCGs, rats have to be immunized with both water-soluble and accessible water-insoluble antigens, like those in unwashed PCGs. For the moment it cannot be ruled out that some allergens are specific to PCGs. Motta et al showed by immunoblots that rats sensitized either with pollen or with PCGs exhibited similar responses to water-soluble allergens [10]. Here, we have shown that proteins recognized as allergens by rat IgE may correspond to allergens also recognized by human IgE. Furthermore, by using monoclonal antibodies, we have identified some allergens, like those of group 1, in water-soluble PCG extract, but allergens of groups 3 and 4 were not found (data not shown). The allergens of these 3 groups (1, 3, and 4) were found in water-soluble *Phleum pratense *pollen extract [25]. Some other studies showed that several pollen allergens were present in PCGs, among which include Phl p 4, Phl p 5, Phl p 6, and Phl p 13 [12,13,16]. In the present study we found water-insoluble allergens in *Phleum pratense *PCGs and pollen grains, like in *Dactylis glomerata *pollen [26,29] and weed pollen grains [30,31].

In cellular studies, for the 3 sensitization groups--pollen grains, unwashed PCGs, and washed PCGs--we observed substantial responses that did not correlate with IgE responses. This was also observed by Würtzen et al, [32] who reported that the immune response to Phl p 5 alone--that is, a qualitatively restricted antigen--involved different cytokine circuits as compared with the immune response to the same protein in a pollen extract, IL-5 versus IFN*γ*, respectively. Similarly, the collection of antigens is qualitatively restricted in washed PCGs as compared with that in pollen grains and the IL-5 level was high in the BALF of rats sensitized to washed PCGs, whereas IFN*γ *was high in pollen-sensitized rats.

In the lymph node cells in vitro experiments, the differential responses for the rats sensitized to washed PCGs and the pollen-sensitized rats suggest that responder T-cell precursors specific to water-insoluble antigens of PCGs are present in bronchial lymph nodes, but that they are not stimulated when the PCGs are packed and/or displayed within the pollen grain. The poor proliferation, of the lymph node cells from unwashed PCGs sensitized rats, in response to pollen and PCGs may be due to differences in the kinetics of cellular immune responses, whereas systemic humoral IgE immune responses were optimally expressed. Such variations in immune response kinetics, monitored by in vitro induced cell proliferation, have been reported to be immunogendependent [33].

In the lung, cell analysis of BALF showed that, whatever the sensitization, macrophages, neutrophils, and eosinophils were equally elevated. For lymphocytes and eosinophils, similar results to ours have been reported in mice sensitized with subpollen particles [8]. Local recruitment of eosinophils and lymphocytes depends on T_H_2 cytokines IL-4, IL-5, IL-13, chemokines GM-CSF, and Rantes, [34-36] which are specifically increased in rats sensitized to washed PCGs (not done for IL-4). Conversely, in BALF of pollen-sensitized animals, T_H_2 cytokines are low and IFN*γ*, a prototypic T_H_1 cytokine, is high, but absent in rats sensitized to washed PCGs. Although LPS is known to promote the production of IFN*γ*, [37] the differential response of rat groups is unlikely to be related to contaminant LPS in sensitizing immunogens. First, TNF*α *and IL-6 responses, both reported to be increased by LPS, [38,39] were similar in groups sensitized with LPS-containing immunogens (pollen and unwashed PCGs) or immunogens not containing LPS (washed PCGs), and second, neutrophils, also reported to increase upon LPS stimulation, [40] are equally elevated in rats sensitized to washed PCGs (LPS-free) and pollen-sensitized rats (LPS-positive). The presence of eosinophils, lymphocytes, and Rantes in BALF was reported to be associated with severe asthma[41-45] and Badorrek et al reported that PCGs induce asthma symptoms whereas whole pollen induces hay fever symptoms, [46] that is, mainly rhinoconjunctivitis. Interestingly, when IgE is elevated, as in the casse of hyper-IgE syndrome, TH17 cells, a cell lineage involved in inflammation, is suppressed [47]. Furthermore, other compounds such as nicotinamide adenine dinucleotide phosphate-oxidase (NADPH-oxidase), fatty acids, and 1, 3-*β*-glucan, present on pollen and PCGs, especially in water-insoluble fractions, enhance nonspecific inflammation [8,48-50]. It should be noted that, depending on where the particles, pollen grains, or PCGs end up in the respiratory tract, the inflammatory impact may not have the same consequences.

In conclusion, we studied several parameters to decipher the inflammatory and immune response in rats to *Phleum pratense *pollen grains and their subfraction PCGs, with a special emphasis on water-soluble and -insoluble proteins: (i) specific IgE production, globally and versus proteins separated in IEF, (ii) cell distribution in BALF, (iii) alveolar cytokine production, and (iv) bronchial lymph node reactivities. Altogether, our results underscore differential T_H_2 responses that confer on the intrinsic proteins of PCGs--that is, water-insoluble proteins--a role in the central cell-mediated inflammatory response and on water-soluble proteins prematurely released from pollen grains a role in the peripheral humoral response. Submicrometric particles of pollen, like PCGs, should thus be considered as an allergenic source with a high pathologic impact. Experiments are in progress to characterize these allergens.

## End note

Supported by the French Ministry of Environment and Sustainable Development (BCRD-DRC-05-AP-2005).

Presented at "Allergenicity of Pollen Grains and Pollen Cytoplasmic Granules", World Allergy Congress, December 2007, Bangkok, Thailand; "Characterization of Water Soluble and Water Insoluble Allergens of Pollen Cytoplasmic Granules, " European Academy of Allergy and Clinical Immunology Congress, June 2008, Barcelona, Spain; and Les granules cytoplasmiques du pollen sont-ils responsables de l'allergénicité des grains de pollen ?" - Congrès Francophone d'Allergologie, April 2008, Paris, France.
